# Young children and families’ home literacy and technology practices before and during COVID-19

**DOI:** 10.1177/1476718X231164132

**Published:** 2023-05-15

**Authors:** Naomi T Lin, Monica Molgaard, Alison Wishard Guerra, Shana Cohen

**Affiliations:** University of California San Diego, USA; University of California San Diego, USA; University of California San Diego, USA; University of California San Diego, USA

**Keywords:** COVID-19 pandemic, early childhood, home learning practices, literacy, technology

## Abstract

The home is an important setting for young children’s learning and development. We examined home literacy practices and technology usage among families with children ages 5–6 years old (*N* = 47) before as compared to during COVID-19 with bivariate analyses. Variations by household income were also investigated. Parents completed surveys on home literacy and technological practices and demographic information in the spring of transitional kindergarten and the following kindergarten year. Results demonstrated an increase in child technology usage for school related educational programs, from pre-COVID-19 to COVID-19 and a decrease in home book reading, storytelling, writing, and playing games during COVID-19 home learning. Transitional kindergarten parents from the upper-income bracket reported significantly lower technology use of educational games compared to those in the lower-income group pre-COVID-19. Kindergarten parents from the upper-income bracket reported significantly higher technology use of educational games and lower technology use in watching TV to fall asleep, compared to those in the lower-income group during COVID-19. Findings suggest that there are few differences in home learning environments across family income groups. By characterizing how parents utilize technology and literacy practices with their children, we can better understand how to support families through COVID-19 and beyond.

Home learning environments are crucial in early child development as children first learn from their interactions at home (Rogoff et al., 2007; Weisleder and Fernald, 2013). In the spring of 2020, school-based learning suddenly shifted to virtual at home learning, dramatically impacting the daily home learning routines by integrating technology mediated school learning routines into the home context (Bao et al., 2020; Wang et al., 2020). In this study, we investigated how home learning practices changed for young children during the COVID-19 mandated stay at home orders. We specifically focused on literacy and technology home learning practices, as children’s technology and literacy fluency are significant predictors of academic outcomes (Gutiérrez and Rogoff, 2003; Ma et al., 2016).

We drew from ecocultural theory to examine the home learning practices of young, socio-economically diverse children in a Southern California school district. The ecocultural niche framework situates learning and development within families’ daily routines and activities, where routines serve to transmit and adapt cultural practices, beliefs, and experiences to individuals (Weisner, 2002). The ecocultural niche, or the family environment, is the space where daily routines and activities are practiced, internalized, and imbued with cultural notions of broad concepts (e.g. education, disability) (Vélez-Agosto et al., 2017; Weisner, 2002). For example, how families use technology at home may mediate children’s learning and home literacy practices. Children’s daily routines may also provide rich contexts for families to build upon cultural understandings of socio-emotional phenomena (e.g. bien educado) and optimize children’s development; this has been shown in studies of Mexican heritage families and their young children (Bridges et al., 2012, 2022).

This framework is useful for studying home learning activities during COVID-19 school closures when all academic learning was happening at home and often integrated into daily routine activities (e.g. brushing teeth, mealtime). For low-income families living in poverty, the ecocultural niche can identify the proximal protective factors, such as parental engagement, that could mitigate risk for children experiencing poverty (McWayne et al., 2016). During initial COVID-19 stay at home orders, school children were required to engage in technology-mediated schooling from home. Ecocultural theory provides a lens to better understand how variations in family routines such as book reading, story telling, and writing activities create the fabric of young children’s home learning environments that shape children’s cognitive and social emotional development. This will in turn provide a nuanced understanding of how families interact, how cultural practices are integrated into these literacy activities, and how to incorporate literacy and language into other daily routines to promote learning opportunities.

## Home learning practices

Previous studies have demonstrated that young children learn through routine everyday interactions in their primary contexts of home and school (Rodriguez and Tamis-LeMonda, 2011; Weisner, 2002). Young children are actively constructing knowledge at home through conversations with their caregivers and siblings or peers. For example, they solve conflicts, have arguments, and read books. There is a reliable and strong positive association between home practices and learning outcomes (Ma et al., 2016). Frequent book reading combined with detailed and engaged conversations about the books predicts literacy outcomes for young children, even after controlling for socioeconomic status (Hartas, 2011; Raikes et al., 2006). Furthermore, longitudinal studies show that the quality of early home learning environments that include enrichment activities and parent child interactive learning processes predict later high quality of home learning environments (Anders et al., 2012; Toth et al., 2020). High quality early home learning environments, often defined by frequent book exposure and complex verbal interactions with adults, help build early language and literacy skills, which are shown to predict academic skills through high school (Lehrl et al., 2020). These studies demonstrate the specific pathways by which activities in early home learning environments support children’s concurrent cognitive and later academic skills, making the study of home learning environments critical to early childhood development.

### Parental engagement at home

There is quite extensive literature on the importance of parent engagement in early childhood and its impact on socioemotional and cognitive development, as well as academic achievement (e.g. Hoover-Dempsey et al., 2005; Hurwitz et al., 2015). Prioritization of social emotional and academic learning goals may vary across socioculturally diverse families, contributing to variations in daily household routines at home (Rogoff et al., 2007). For example, Reese and Goldenberg (2008) found that Spanish speaking immigrant families in the United States tended to utilize language learning opportunities, such as oral conversations, rather than the traditionally academic practices, such as books and other printed materials, to facilitate language development in their bilingual children. In addition, family socioeconomic status is a known correlate with daily routines, such that parents’ schooling and literacy practices are associated with their home literacy practices with their children (Reese and Goldenberg, 2008). Past research has shown that children from lower-income families spend more time in unstructured and independent activities, while children from upper income families spend more time in adult-organized activities specifically focused on academics (Lareau and Weininger, 2003). Lareau suggests that variations in daily activities transfer differential values and advantages to children, not necessarily better or worse. She argues that children from upper-income families may be better prepared for school as a result of their daily activities and family ecology, while children in middle to lower-income families may be more comfortable making their own decisions and forming and sustaining relationships with extended family and social networks.

Parent engagement is a substantial to creating a home environment that is beneficial for the child’s development. Home environments are extremely important in early learning as they seem to relate to the outcomes of early schooling, specifically pre-kindergarten to kindergarten (Ansari et al., 2020; Han et al., 2020; Karoly, 1998). The sustainability of pre-kindergarten positive outcomes, such as letter word identification and vocabulary development, are more likely when there is a higher quality of home learning environment due to the continued engagement of academic and language interactions (Ansari et al., 2020; Han et al., 2020). Since there are few studies looking at long term early schooling results during the pandemic, it is increasingly crucial to examine how home learning environments may benefit children’s early education.

### COVID-19 impacts on home learning practices

As a result of the onset of COVID-19, virtually all children in the United States and worldwide had face-to-face instruction interrupted during the 2019–2020 school year. To stop the spread of COVID-19, 130,000+ schools closed, impacting approximately 57 million children who were ordered to stay at home (Bao et al., 2020). These school closures prompted schooling to occur at home, while many parents also had to shift to working from home. These major life changes forced parents to balance the roles of teaching, working, and parenting. Interactions between parents and children increased (e.g. families argued and interacted more, children and parents played together more), resources at home became the only resources children had access to, and social interactions between same age groups diminished (Duran and Ömeroğlu, 2022; Garbe et al., 2020; Mantovani et al., 2021)

Researchers and educators anticipate significant long-term impacts on children’s developmental and academic outcomes (Bailey et al., 2021; Kuhfeld et al., 2020; McCoy et al., 2021). McCoy et al. (2021) utilized simulation methods applied to pre-pandemic data from UNESCO from 196 countries to estimate the implications of COVID-19 related early childhood school closures for children’s short- and long-term outcomes. Prior to COVID-19, 84 million children are developmentally off track and an additional 10.75 million children may become off track, according to the simulation model (McCoy et al., 2021). Kuhfeld et al. (2020) projected that students will not have shown as much academic growth during their time physically out of school due to the variation in parent and teacher supports. However, their study also projects that the students who lost the most while outside of school would gain the most once back in school. While there are many factors that contribute to achievement, Kuhfeld et al. (2020) study provides some hope that students affected most will be able to rebound.

The impacts of COVID-19 have further widened income-based achievement gaps between high- and low-income parents, particularly with their ability to support their children academically (Bailey et al., 2021). Low-income families are more likely to attend public schools that lack the resources necessary in supporting teachers’ efforts in providing high-quality instruction through online platforms (Hamilton et al., 2020). Additionally, low-income families are less likely to have access to high-quality internet and computers in their homes, making it challenging for children to participate in their online schooling. Schools in low-income areas may not have enough computer devices for each individual child; therefore, families with multiple children have one device to share (García et al., 2020). This may lead to situations where children in the household rotate days where they are able to access their online instruction. Low-income families are also less likely to be able to supplement the online instruction their children are receiving with various forms of enrichment or private tutoring (Kniffin et al., 2021). If these families deem the instruction their children are receiving from the public school to be inadequate, they are less able to switch schools and enroll their child in a private school.

## Literacy home practices

A crucial component to home learning environments home literacy practices. Home literacy practices refer to interrelated language and literacy experiences that occur at home such as book-reading (shared and individual), storytelling, writing, and extended conversations between caregivers and children (Dickinson and Tabors, 2001; Guo et al., 2021). Oral language interactions are the foundations of formal literacy development, with substantial evidence linking variations in home language and literacy practices to school outcomes (Dickinson and Tabors, 2001; Fernald et al., 2013; Hart and Risley, 1995). Having conversations with their parents, siblings, and others at home and growing up in an home environment with higher frequency and complexity of oral language leads to higher vocabulary, verbal, and reading comprehension skills compared to those growing up in less verbose households (Gardner-Neblett et al., 2012; Hart and Risley, 1995; Tenenbaum et al., 2005). These home language interactions are important as they predict the quality of home learning environments along with academic outcomes (Lehrl et al., 2020; Toth et al., 2020). Home literacy practices are also known to be culturally informed and enacted in diverse ways across socioculturally diverse families. While income based disparities in home language environments are linked to parallel disparities in oral language skills in early childhood and formal literacy skills later in elementary school (Fernald et al., 2013; Hart and Risley, 1995), a narrow operalization (i.e. socioeconomic factors) of home language practices conceals culturally relevant adaptive practices that may promote critical language development (Avineri et al., 2015). Among dual language learners, research has pointed to the important and differential impact of home language and literacy experiences on language development. Lewis et al. (2016) found that while frequency of book reading was positively related to children’s Spanish language skill and frequency of storytelling influenced their performance on English oral language measures. Research focused on immigrant Latinx families has described how families modify their own cultural model of literacy by integrating school-based literacy activities into daily routines to aligned with their goals for their children’s academic success (Perry et al., 2008; Reese and Gallimore, 2000).

### Home literacy practices during COVID-19

The effect that school closures have on literacy skills has been previously studied in the closure of schools during the summer months. Studies that explored the effects of summer on children’s academic skills can be utilized to gage COVID-19 school closures effects on children’s skills, though COVID-19 school closures are more severe. Studies have well documented that children lose the equivalent of approximately 1 month of literacy skills during summer break (Bao et al., 2020). There was also a significant decrease in adults reading with children during the pandemic (Read et al., 2022). Children who do not enjoy reading or have access to limited books are less likely to read during the summer, leading to a further loss in literacy skills (Guo et al., 2021). This literacy loss can be reduced by children participating in literacy-rich activities, such as visiting libraries, attending summer school, or engaging in enrichment activities and classes; however, during the mandated school closures, these activities were not available so alternatives that will reduce literacy loss within the home environment must be discussed (Bao et al., 2020).

Bao et al. (2020) found that reading daily to children at home could mitigate literacy loss during COVID-19, while also strengthening family bonds. Young children depend on adults to provide them access to books by reading them aloud, which provides children with high sentence complexity and vocabulary and provides an opportunity to practice the forming of words with the interaction of letters and sounds. However, not all children have access to high quality books or an adult at home who can read them aloud. Parents in low-socioeconomic families are more likely to be “frontline workers”; therefore, they may not have the capacity to support their children academically, compared to higher-socioeconomic families (Bailey et al., 2021). However, research also demonstrated different parental support and involvement by low-income families, such as oral storytelling (Billings, 2009; McWayne et al., 2008).

## Using technology in home learning practices

As learning is occurring at home, it is necessary to focus on what tools are mediating children’s learning. Technology is a tool that is increasingly influencing early child development at home as children watch TV, play video games, play educational games on their iPads on a daily basis (Dorouka et al., 2020). For example, children, especially those from low- and middle-income families, who watched Sesame Street demonstrated improvements on their literacy and numeracy outcomes, social reasoning and skills, and understanding of the world, such as health and safety (Mares and Pan, 2013).Through the many outlets of technology, children can learn language, problem solving, and even social skills through TV shows, games, and interactions with others during the technological usages like messaging and other mobile applications (Oh and Jonassen, 2007; Weinberger et al., 2007). Research has shown the effectiveness of learning with technology as children are engaged with technology tools in classrooms and develop problem solving skills (Marsh et al., 2018; Yen and Lee, 2011). Specific mobile programs had positive effects on literacy development, mathematics, science, problem solving, and self efficacy for children ages 2–5 due to the interaction basis of these programs to engage children with new information, as well as further develop their transfer, working memory, and other cognitive skills (Herodotou, 2018). Additionally, with increasing technological access, children are more connected to current culture and media as well as overall understandings of the digital world (Marsh et al., 2005). However, not all children have the same access to technology (Tawfik et al., 2016). While some children have a plethora of online learning resources, some children may not. Depending on the socioeconomic status of students, teacher’s age and education, and the programs, there are significant access barriers to successful learning with technology for students (Blackwell et al., 2013; Tawfik et al., 2016). Moreover, the design of technological tools may also have unintended consequences for students and families who lack user experiences (Tawfik et al., 2016). Some children who are engrossed in technology usage may also be lacking in play and outdoor and social activities that may be helping other aspects of socioemotional, motor, cognitive development (Plowman et al., 2010).

### Home technology practices during COVID-19

As school shifted to online, technology became a crucial tool to connect school to home as children attended Google classrooms and Zoom sessions (Edwards et al., 2018). Prior to COVID-19, technology may not have had as prominent of a role in home learning practices; children now rely on technology to learn from school. Children who did not have access to technology now have iPads or Chromebooks provided by the school and parents are now gaining knowledge of how to use various forms of technology to support their young children (Andrew et al., 2020).

As there is a positive and strong correlation between learning outcomes and parental involvement, it is also important for school and home environments to be aligned to better support the academic and cognitive development of young children. The family and school connections are related to the development of socioemotional, language, motor, and cognitive development. By knowing how home environments change and impact learning outcomes, this can help teachers in a post-COVID-19 environment to better understand home learning and build on family practices and capacity, and align with school practices. By characterizing their everyday learning environment, we will examine home literacy and technology practices in both higher and lower-income families before and during school closures forced by the COVID-19 pandemic. Our study explored how home learning practices differ between pre-COVID-19 pandemic and during the first year of the COVID-19 pandemic. The following research questions are addressed:

1. How did home literacy practices and technology usage with families of children ages 5–6 years old during the COVID-19 mandated stay at home orders differ from home practices prior to COVID-19 stay at home orders?
2. How did home literacy and technology practices vary by family annual household income prior to COVID-19 and during COVID-19?

## Methods

### Design

#### Larger study context

The subset of our data is from an overarching 3 year longitudinal Research Practice Partnership (RPP) project in a medium sized socioeconomically diverse school district in Southern California where 61% of students received free and reduced lunch in 2018–2019, studying the early cognitive, social emotional and academic development of early elementary school aged children (see Wishard Guerra et al., 2020 for a detailed description of the larger study design). Transitional kindergarten (TK) is the first year of public elementary school in California serving older 4 year old children who were born between September 1 and December 1 and provides younger students with “a modified kindergarten curriculum that is age and developmentally appropriate” defined by California law (EC 48000) (California Department of Education, n.d.). All TK teachers in the district were recruited to participate in the RPP project to collaborate with researchers in identifying urgent problems of practice and co-design a research study to help inform these timely problems. All families from participating TK classrooms (*N* = 6) were then invited to attend information sessions with the lead researchers to learn more about participation in the project. After presenting information about the project and explaining the activities that children and parents would be invited to participate in, an average of 42% of families in each classroom consented to participate. Participating families had an average of 4.5 (range 2–10) people living in the home, an average of two adult wage earners per household (range 1–6), with a median household income of $50,000–$75,000 per year. For this portion of the study, we used an exploratory sequential mixed methods design with Qualtrics online survey measures collected from each family annually. We characterized the overall home learning environment with the survey data from year 1 pre-COVID-19 and year 2 during COVID-19.

#### Participants

Forty-seven families, with children (mean age in years: 5.56, 48.9% female, 46.2% Hispanic) in TK through kindergarten (K), participated in the first year (pre-COVID-19) and 36 families participated in the second year (COVID-19) parent surveys. In our study, there were two sets of twins and 19.6% of the child participants spoke Spanish as their first language. The surveys were completed by the child’s biological parent or legal guardian.

### Materials and procedures

The project was approved and overseen by the school district’s superintendent and participating teachers. The children’s parents voluntarily participated in full informed consent procedures, renewed annually, and the children participated voluntarily and gave assent. Families were remunerated for their participation in each research activity. To address potential ethical concerns about breach of confidentiality when sharing child or family level data back with the district or with a wider academic audience, all data were de-identified to protect child and parent confidentiality and presented back to the school district staff in aggregate form. Each year, families were invited to participate in an annual online survey to collect demographic information on their ethnicity, home language, and family income. Income was grouped into upper and lower-income by the California low-income threshold of $50,000. Two families did not answer the household income question. We determined the lower household income families by the qualifying California low household income threshold of $50,000. Lower-income households are defined as having under $50,000 overall income and higher income households are over $50,000. There were 34.3% in lower-income households in TK and 28.6% in K. We were interested in the caveat of the lower-income grouping decreasing in the pandemic year of K. When analyzing the household income, with hierarchical cluster analysis instead of California low threshold grouping, the two average lower-income groups were different. In TK, the low-income group reported an average household income of $25,001−$35,000. The upper-income group reported an average income of $75,001−$100,000. In K, the low-income group reported an average income of $15,001−$25,000; the upper-income group reported an average income of $75,001−$100,000. The lower-income groups in K decreased from $25,001–$35,000 group to $15,001−$25,000. Although this is not captured in our overall analysis of lower and upper household income threshold groups with the California low household income threshold, it is important to note that lower-income households were affected greatly by the pandemic.

Home literacy practices and technology usage were collected with the parent survey sent out each year in spring. We asked parents about their frequency in engaging in the following activities using a 4-point Likert-type scale ranging from rarely or never to daily: Read books with your child, Tell stories to your child, Write with your child, Cook with your child, and Play games with your child. Using a 5-point Likert-type scale, ranging from never to 4 or more hours, parents also rated their children’s engagement with technology on use of technology for education, use of tablet/smart phone, entertainment for fun, watching TV or movies, and to assist with sleeping.

## Results

### Overall descriptors of home environment measures in TK and K

Parents answered home environment surveys when their child was in TK during pre-COVID-19 and in K during COVID-19. Descriptive statistics of home literacy and technological practices are reported in Table 1. We compared the home practices from pre-COVID-19 (*N* = 47) and COVID-19 (*N* = 36–40, due to incomplete survey questions) with paired two tailed *t*-tests. There was an increase from pre-COVID-19 to COVID-19 in child technology usage for school related educational programs (t(35) = 2.84, p = 0.008). Parents reported less than 1 hour per day for educational programs during pre-COVID-19, which increased to 1–2 hours per day during COVID-19. During K COVID-19 time, there was a decrease in home literacy practices of book reading (t(35) = 3.75, p = 0.001), storytelling (t(33) = 3.97, p = 0.004), writing (t(34) = 5.94, p < 0.001), and playing games (t(34) = 2.94, p = 0.006). Parents reported they read books, told stories, and wrote with their children five to six times per week pre-COVID-19 and two to four times per week during COVID-19.

**Table 1.** Descriptives of TK and K home literacy and technological practices and paired *t*-test.

Home Practices	*TK*	*K*	*t*
	*M* (SD)	*M* (SD)	
Read books with child	3.11 (0.81)	2.62 (1.0)	−3.75**
Tell stories	2.74 (0.87)	2.34 (0.85)	−3.97**
Write with child	2.7 (0.75)	2.03 (0.71)	−5.94**
Cook with child	2.26 (1.01)	2.13 (0.77)	−0.70
Play games with child	3.21 (0.75)	2.77( 0.84)	−2.94**
Educational programs/apps from school	2.36 (0.94)	2.88 (0.88)	2.84**
Games on tablet or smartphone	2.91 (0.97)	2.54 (0.99)	1.60
Educational games for fun	2.28 (0.85)	2.5 (0.81)	0.85
Watch TV or movies	2.74 (0.68)	2.89 (0.74)	0.89
Watch TV to Fall asleep	1.28 (0.68)	1.33 (0.63)	0.96

** *p* < 0.01.

### Differences between home practices with TK and K lower and upper household income groups

To determine if there were significantly different home literacy and technological practices between the upper and lower household income groups, we conducted one-way ANOVAs. In TK, parents from the upper-income bracket reported significantly lower use of educational games, *F* = (1, 45) = 5.43, *p* = 0.02, when compared to that of parents in the lower-income bracket. In K, parents from the higher-income group reported significantly higher technology usage of educational games for fun, *F* = (1, 34) = 7.20, *p* = 0.01, and significantly lower technology usage in watching the TV to fall asleep than those in the lower-income group, *F* = (1, 34) = 9.34, *p* = 0.004. There were no other significant differences by family income group.

## Discussion

Past research demonstrated the importance of home learning practices for early education and academic development (Gutiérrez and Rogoff, 2003; Ma et al., 2016; Rodriguez and Tamis-LeMonda, 2011). Children’s social interactions with family members as they engage with various literacy and technological tools at home are essential in shaping the development of children’s cognitive skills. Furthermore, when exploring the early learning and development period, it is necessary to examine the integrative cultural process of home learning. The daily home practices are intertwined with how the children and their families learn in various sociocultural contexts, such as how their learning environment is physically structured, what tools they use for learning, and what dialog is used in communicating with family members (Rogoff et al., 2007; Weisner, 2002). The pandemic has been a forceful change for families with young children as schools transitioned to the unprecedented format of distant learning, affecting early education drastically (UNESCO, 2020; World Bank, 2021). Teachers and families had to learn how to use technology, communicate with each other online, and help children adapt to learning virtually. As parents, teachers, and young children are all adjusting to this time of great adversity, the home environment, specifically technology and home literacy practices, is significantly different from the pre-pandemic era. We found significant decreases in parent reports of the daily practices of reading books, writing, and telling stories, along with playing games, in our participating families’ homes. In addition to children not receiving the same dosage of teaching and learning from school, they also experienced a decrease in reported frequency of home literacy activities. These changes at home may be attributed to parents simultaneously dealing with COVID-19 effects on their professional and personal lives, thus affecting their abilities to fully be caregivers, parent-teachers, and working professionals (Spinelli et al., 2020). As COVID-19 impacted home routines, and thereby home learning practices, families’ support for their children’s learning also changed. With better support for families from the schools, beyond COVID-19, parents integrating daily literacy practices would be less stressful than “adding” more responsibilities for the parents to do. Daily practices are a way for parents to easily increase their children’s learning opportunities in literacy. As an example of integrating daily literacy practices, parents enriched their children’s daily literacy practices by storytelling during breakfast, talking during car rides, and reading books during bedtime.

Another possibility for these changes in home literacy practices could be attributed to diverse families’ backgrounds and cultural practices (Reese and Gallimore, 2000). Although this study did not include an analysis of variations by cultural background, past research has shown that different cultures integrate literacy practices in various ways such as oral story telling rather than reading physically printed sources (Reese and Goldenberg, 2008) and these culturally anchored practices may have been differentially impacted by families’ diverse experiences with COVID-19 stay at home orders. The quality of home learning environments may also be associated with the family’s resources and age of children as with older children, family involvement in home learning environments decreases (Blaurock and Kluczniok, 2019). Although families are adapting during an unprecedented time, these changes in literacy and home practices can be normal.

Our second research question examined home literacy and technology practices differences between family income. Existing research has shown variations in home learning practices between families living in poverty and families in middle or upper income brackets (Lareau and Weininger, 2003; Magnuson and Duncan, 2002). Contrary to these findings, our data indicated minimal differences in the frequency of home learning routines by income group. All families engaged in home literacy and technological practices known to support academic development, both before and during COVID-19 school closures. Low-income families engaged in equivalent levels of reading, writing, and storytelling when compared to upper-income families. This finding challenges the deficit notions that families experiencing poverty or less educated parents do not have the resources or habits to support their children’s learning (Lareau, 2011).

When virtual learning began when schools shut down during COVID-19, children were provided with technological tools, such as iPads and other online curriculum and educational programs (Raz Kids, 2002; ST Math, 1998) funded by the school (Andrew et al., 2020). With our study sample, there was a significant increase in educational programs usage from pre-COVID-19 to COVID-19 as technology became a necessity for children to learn. These educational programs can be utilized while in virtual classrooms as well as asynchronous learning. Not only do the children now have these additional resources at home, but also young children are quickly acclimating to the concept of learning independently (Dorouka et al., 2020; Mares and Pan, 2013). One concern is related to the higher frequency of technology during bedtime among lower-income families. Some concerns about the overuse of technology include lack of rest or socioemotional development (Plowman et al., 2010). Low-income families may be challenged with housing instability and limited space for children to have protected quiet time at bedtime, as rest is important for development and for the children to have energy the next day. Parents may be able to use technology in an effective way such as audio bedtime or meditation stories. With these challenges more present, parent education about alternative ways to support winding down at bedtime (bedtime stories, going to bed at a consistent time, etc.) could benefit children and families, such as reducing anxieties, especially during the COVID-19 era (Ferretti and Bub, 2017).

As home environments are increasingly aligned with at home school learning, educators can build on the strengths of family home learning practices to support academic development. Although these findings are comparing pre-COVID-19 and COVID-19 home literacy and technology practices, the longitudinal aspect of these findings can be useful in informing how families cope with changes at home as well as the normal shifts of home learning practices. Children are both learning and living culture at home, making home practices an essential element of understanding child academic and cognitive development (Gutiérrez and Rogoff, 2003). With virtual schooling, more children are provided with more equal learning opportunities at home, such as devices and educational programs supplied by the school. As educators further strengthen the alignment between home learning and school curriculum learning, young children can benefit from learning in the home space with parents as teachers. This may be useful in lifting academic trajectories and reducing achievement gaps with home learning.

## Limitations/implications for research and practice

There are several limitations to the current study; first, though our participants were linguistically and socioeconomically diverse, the sample size was relatively small. Second, the data is based on retrospective reports by parents, which limits the data to their perceptions of how the home learning practices have changed and may not have been the actual home practices changes. Furthermore, we didn’t specify which aspects of literacy or technology practices were teacher supported at home. Third, our study sample was from a particular region of Southern California, which may not be generalizable to other geographic regions in the United States or globally. As families can effectively change their learning practices in their own home, it is important to identify strategies in supporting home practices, specifically cultural responsive support for families and children to learn effectively. Future work should include a larger sample size from a linguistically, culturally, and socioeconomically diverse population for the findings to be more broadly generalizable. Additionally, further research on more detailed measures of literacy and technology, such as family interviews or observations, are needed to make substantial implications of COVID-19 home literacy and technology differences amongst lower and upper income groups. As the study is longitudinal, it will be extremely valuable to examine the home environment after a year in the pandemic.
